# Revisiting the clastosome: a stress-induced nuclear proteolytic compartment of mammalian cells

**DOI:** 10.3389/fnana.2026.1839944

**Published:** 2026-06-02

**Authors:** Miguel Lafarga, María T. Berciano, Noemí Rueda, Sofía Cardona-Cortés, Olga Tapia

**Affiliations:** 1 Department of Anatomy and Cell Biology, University of Cantabria, Santander, Spain; 2 “Centro de Investigación en Red sobre Enfermedades Neurodegenerativas” (CIBERNED), Research Group CB06/05/0037, Santander, Spain; 3 Department of Physiology and Pharmacology, University of Cantabria, Santander, Spain; 4 Department of Basic Medical Sciences, Institute of Biomedical Technologies (ITB), Universidad de La Laguna (ULL), Tenerife, Spain

**Keywords:** cellular stress, clastosomes, down syndrome, mammalian neurons, nuclear condensates, nuclear proteolytic centers, PML nuclear bodies, ubiquitin-proteasome system

## Abstract

The nuclear ubiquitin proteasome system (UPS) is fundamental to maintaining proteostasis and ensuring the quality control of nuclear proteins. Within the nucleus, proteasomes are distributed throughout the nucleoplasm and can aggregate into nuclear bodies. In 2002, our group provided the first description in mammalian cells of a specific subtype of nuclear body highly enriched in: (i) ubiquitin conjugates, (ii) the proteolytically active 20S and 19S regulatory complexes of the 26S proteasome, (iii) the molecular chaperone Hsp70, and (iv) proteasome substrates. We coined the term clastosome to define this nuclear proteolytic center of the UPS. Clastosomes exhibit dynamic behavior, and their formation is transiently and robustly induced in mammalian neurons during osmotic stress, coinciding with enhanced proteasomal activity. Subsequent studies have confirmed and mechanistically expanded our understanding of these nuclear proteolytic centers, which are now recognized as nuclear condensates formed via liquid–liquid phase separation mechanisms. Notably, PML nuclear bodies can establish interactions with clastosomes, thereby linking the PML protein and the SUMOylation pathway with UPS-mediated protein degradation. The association between PML nuclear bodies and clastosomes has been implicated in the clearance of toxic mutant proteins associated with certain neurodegenerative diseases. This review examines the structure, composition, and dynamic regulation of clastosomes, focusing on their modulation during stress responses and neurodegeneration. Given the essential role of nuclear proteolytic centers in proteostasis regulation, a better understanding of mechanisms that modulate their assembly may offer therapeutic strategies for neurodegenerative proteinopathies and other pathologies.

## 1 Introduction

The interphase nucleus is a highly organized environment characterized by dynamic constitutive or inducible nuclear compartments that orchestrate transcription, RNA processing, nucleocytoplasmic transport, spatial genome organization, DNA damage/repair, and the cellular stress response (Dundr and Misteli, 2001; Cremer et al., 2006; Mao et al., 2011; Staněk and Fox, 2017; Bhat et al., 2021; Belmont, 2022; Shan et al., 2024; Lafarga et al., 2025; Malszycki et al., 2026; Liu et al., 2026). This compartmentalization enables the segregation of functional subnuclear domains, thereby enhancing the efficiency and specificity of metabolic processes within each domain.

Most of these compartments are membraneless nuclear bodies, such as the nucleolus, Cajal bodies, nuclear speckles and promyelocytic leukemia nuclear bodies (PML-NBs), characterized as nuclear condensates assembled via liquid–liquid phase separation (LLPS) mechanisms (Hyman et al., 2014; Sawyer et al., 2019; Fu et al., 2021; Yu et al., 2021; Lyon et al., 2021; Lafontaine et al., 2021; Feric and Misteli, 2022; Mehta and Zhang, 2022; Hirose et al., 2023; Li et al., 2025; Chen and Zhu, 2026; Shinn et al., 2026). These condensates establish microenvironments distinct from the surrounding nucleoplasm by selectively recruiting and concentrating specific molecules. Particularly, they concentrate RNAs and proteins containing intrinsically disordered regions (IDRs), which facilitate multivalent macromolecular interactions (Reviewed in Banani et al., 2017; Hirose et al., 2023).

In the nucleoplasm, the ubiquitin-proteasome system (UPS) is essential for maintaining proteostasis and ensuring the nuclear protein quality control (PQC). Consequently, the nuclear UPS contributes to regulate steady-state protein levels according to dynamic nuclear demands and prevents the accumulation of misfolded or defective proteins (Janer et al., 2006; von Mikecz, 2006; Sampuda et al., 2017; Yasuda et al., 2020; Lulu-Shimron et al., 2025).

A paradigm shift in the understanding of nuclear proteasomes occurred with the identification of transient nuclear bodies that concentrate components of the UPS and proteasome substrates. This discovery supported the model of these structures as sites of nuclear proteolysis (Lafarga et al., 2002a; Carmo-Fonseca et al., 2010; Yasuda et al., 2020; Uriarte et al., 2021; Fu et al., 2021, 2024; Iriki et al., 2023). Lafarga and colleagues originally described a nuclear proteolytic center of the UPS that concentrated active proteasome complexes and proteasome substrates within a discrete nuclear body. The term “clastosome”—derived from the Greek *klastos* (broken) and *soma* (body)—was introduced to designate this nuclear compartment (Lafarga et al., 2002a). In mammals, clastosomes were described in neurons *in vivo* as well as in cultured cells (Lafarga et al., 2002a; Villagra et al., 2008; Baltrons et al., 2008; Carmo-Fonseca et al., 2010; Casafont et al., 2016).

In this review, we delineate the structure, molecular composition, and dynamic regulation of clastosomes in mammalian cells, with a specific focus on their modulation during cellular stress, and neurodegenerative pathology. Moreover, the possible interplay between PML-NBs and clastosomes is also analyzed.

## 2 Overview of the ubiquitin proteasome system

Proteolysis is fundamental to the regulation of protein half-life and PQC. The disruption of proteostasis, leading to the accumulation of misfolded proteins, is associated with the pathogenesis of certain human diseases, including severe neurodegenerative disorders (Ciechanover and Brundin, 2003; VerPlank et al., 2018; Guo et al., 2014; Zbinden et al., 2020; Chowdhury and Enenkel, 2015).

Eukaryotic cells employ two primary systems for protein degradation: the lysosomal and the proteasome-mediated pathways (Coux et al., 1996; Hershko and Ciechanover, 1998; Dikic, 2017). Whereas long-lived proteins are preferentially degraded via lysosomal proteolysis, most intracellular protein turnover—especially that of short-lived, regulatory and misfolded proteins—is mediated by the UPS (reviewed in Chowdhury and Enenkel, 2015). Proteasome substrates include transcriptional regulators, cell cycle factors, oncogene products, inflammatory mediators, and defective or misfolded proteins (Ciechanover et al., 1991; Szutorisz et al., 2006; Guo et al., 2014; Chowdhury and Enenkel, 2015; Fu et al., 2021).

The 26S proteasome is a highly conserved, abundant macromolecular complex essential for the maintenance of eukaryotic proteostasis. It comprises a 20S core particle (CP), flanked by one or two 19S regulatory particles (RPs) (Coux et al., 1996; Finley, 2009; Bard et al., 2018; Finley and Prado, 2020). The 20S CP exhibits a barrel-shaped architecture in which α and β subunits are organized in four heptameric rings. The proteolytically active sites reside in the β1, β2, and β5 subunits, which provide caspase-like, trypsin-like, and chymotrypsin-like activities, respectively. The α subunits harbor nuclear localization signals (NLSs) that facilitate the nuclear import of 20S complexes. The 19S RP, consisting of both ATPase and non-ATPase subunits, mediates the recognition, unfolding, and translocation of ubiquitylated protein substrates into the catalytic chamber of the 20S CP (Coux et al., 1996; Lam et al., 2002; Lu et al., 2015; Arkinson et al., 2025). Spatiotemporal dynamics captured via live-cell imaging suggest that the 26S complex is assembled in the cytoplasm and subsequently translocated into the nucleus through nuclear pore complexes (Pack et al., 2014). In vertebrates, a specialized proteasome composition, the so-called immunoproteasome, contains alternative active sites (β1i/LMP2; β2i/Mecl-1; β5i/LMP7) that enhance substrate turnover relative to standard proteasomes (Seifert et al., 2010; Basler and Groettrup, 2021).

In the early 1980s, seminal research by Hershko and colleagues established that proteasomal degradation typically requires the prior polyubiquitylation of protein substrates. This process implicates the sequential participation of an ATP-dependent enzymatic cascade involving ubiquitin-activating (E1), ubiquitin-conjugating (E2) and ubiquitin ligase (E3) enzymes (Hochstrasser, 1996; Hershko and Ciechanover, 1998; Finley and Prado, 2020; Chowdhury and Enenkel, 2015; Ciechanover and Livneh, 2025). Upon delivery to the proteasome, ubiquitin chains are cleaved and recycled by associated deubiquitinating enzymes (Crosas et al., 2006).

Polyubiquitylated substrates are recognized by the ATPase subunit S6 (Rpt5) of the 19S RP (Lam et al., 2002), unfolded, and threaded through a central passageway into the 20S CP degradation chamber (Finley and Prado, 2020). This process yields short peptides (3–20 amino acids) that are subsequently hydrolyzed into free amino acids by cytosolic aminopeptidases (Kisselev et al., 1998). Notably, certain peptides evade complete proteolysis and are sequestered by MHC class I molecules for antigen presentation (Rock et al., 1994).

Ultimately, the UPS plays a critical role in mammalian cells by modulating proteolytic flux in response to physiological and pathological fluctuations in demand (Steffen et al., 2010). Beyond its role in PQC, the UPS is a central regulator of the cell cycle, gene expression, and various other essential homeostatic processes (Goldberg, 2003; Krogan et al., 2004; Guo et al., 2014; Ahuja et al., 2017; Thapa et al., 2020; Enenkel and Ernst, 2025; Larsen et al., 2026).

## 3 The nuclear proteasomes

Over the past three decades, substantial experimental evidence has supported the presence of proteasomal complexes within the mammalian cell nucleus (Kleinschmidt et al., 1983; Mengual et al., 1996; Brooks et al., 2000; von Mikecz, 2006; Lafarga et al., 2002a; Yasuda et al., 2020; Fu et al., 2021; Guo, 2022; Iriki et al., 2023). Notably, several 20S proteasome subunits possess NLSs that facilitate their active nuclear import (Tanaka et al., 1990; Nederlof et al., 1995). In addition, an array of other UPS components has been localized to the nucleus. They include ubiquitin conjugates, the regulatory subunit of the 20S proteasome PA28γ, the proteasome activator Blm10, the deubiquitinating enzyme HAUSP, and various E1–E3 enzymes such as the SUMO-dependent ubiquitin ligase RNF4 (Lallemand-Breitenbach et al., 2008; Fort et al., 2015; Jonik-Nowak et al., 2018; Fu et al., 2024). Concurrently, an expanding repertoire of nuclear proteasome substrates has been identified (reviewed in von Mikecz, 2006; Guo et al., 2014; Jonik-Nowak et al., 2018; Guo, 2022).

Early investigations employing subcellular fraction immunoblotting, immunofluorescence, and immunoelectron microscopy—utilizing antibodies directed against 19S, 20S, and 26S proteasomes—delineated a dual cytoplasmic and nuclear distribution in mammalian cells (Rivett et al., 1992; Mengual et al., 1996; Brooks et al., 2000). Elevated nuclear proteasome concentrations are frequently observed in highly proliferative cells, tumor cells, and neurons, although precise spatial partitioning varies according to cell type, cell cycle phase, and metabolic activity (Rivett et al., 1992; Mengual et al., 1996; Lafarga et al., 2002a; Zhou et al., 2023; Enenkel and Ernst, 2025).

**Figure 1:**
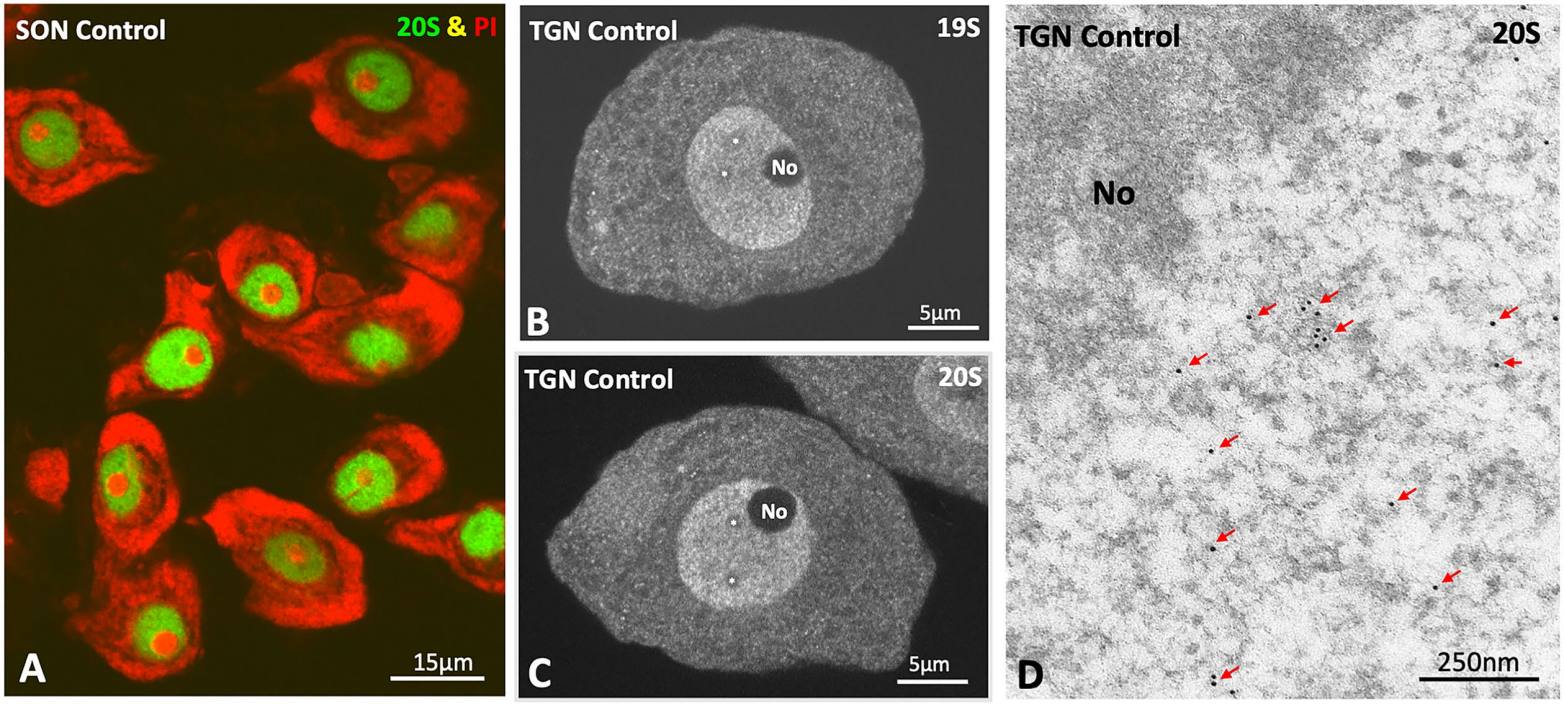
**(A)** Double fluorescent labeling of the 20S proteasome and propidium iodide (PI) in mechanically dissociated supraoptic neuron (SON) cell bodies from a control rat. Note the high nuclear concentration of the 20S proteasome. Structures, enriched in rRNAs, namely nucleoli and polyribosome-rich cytoplasmic areas (Nissl substance), appear intensely contrasted with PI. **(B,C)** Immunolabeling illustrating the nuclear and cytoplasmic distribution of the regulatory 19S and catalytic 20S proteasomes in rat trigeminal ganglion neurons (TGN). Note the higher concentration of proteasomes within the nucleus, the existence of nucleoplasmic domains with reduced proteasome signals (white asterisks), and the absence of both 19S and 20S proteasomes within the nucleolus (No). **(D)** Immunogold electron microscopy detecting the 20S proteasome and showing its localization in euchromatin regions (red arrows). The nucleolus (No) lacks proteasome signal.

Within the nucleus, proteasomes exhibit a diffuse yet non-homogeneous distribution. They preferentially associate with euchromatin while remaining largely excluded from the nucleoli and nuclear envelope (Figures 1A–D) (Mengual et al., 1996; Rockel and von Mikecz, 2002; Lafarga et al., 2002a; Wójcik and DeMartino, 2003; Rockel et al., 2005). Additionally, in cultured cells, proteasome complexes have been found to colocalize with nuclear speckles (Rockel et al., 2005; von Mikecz, 2006; Baldin et al., 2008). Live-cell imaging of GFP-tagged proteasome subunits confirmed enrichment in euchromatin regions and exclusion from the nucleolus. Moreover, fluorescence recovery after photobleaching (FRAP) analysis have demonstrated that while proteasomes diffuse rapidly within both the cytosol and nucleoplasm, their translocation from the cytoplasm into the nucleus occurs at a significantly slower rate (Reits et al., 1997). The presence of catalytically active nuclear proteasomes has been further validated using fluorescence correlation spectroscopy (Pack et al., 2014).

The functional scope of nuclear proteasomes has expanded significantly in recent years. These complexes are now known to participate in: (i) nuclear PQC (von Mikecz, 2006; Borgert et al., 2022; Lulu-Shimron et al., 2025); (ii) regulation of mitotic and meiotic cell cycles (Glotzer et al., 1991; Lafarga et al., 2002b; Craney and Rape, 2013; Ahuja et al., 2017); (iii) control of gene expression through modulation of transcriptional regulators and chromatin remodeling (Szutorisz et al., 2006; Vaughan et al., 2021; Larsen et al., 2026); (iv) nuclear mRNA processing (Thapa et al., 2020); and (v) DNA damage repair (Krogan et al., 2004), among others. These processes are further refined by nuclear-specific proteasome activators and regulators, such as PSME3/REGγ and Blm10 (Qian et al., 2013; Fort et al., 2015; Jonik-Nowak et al., 2018).

## 4 The clastosome: a nuclear proteolytic center

The existence of clastosomes was initially reported in mammalian neurons *in vivo* and in cultured cell types (Lafarga et al., 2002a). The authors demonstrated that clastosomes concentrate active 19S and 20S proteasomes, specific proteasome substrates (e.g., c-Fos and c-Jun), and the molecular chaperone Hsp70. This structure was proposed to function as transient, specialized proteolytic hubs of the UPS (Lafarga et al., 2002a; Carmo-Fonseca et al., 2010). In fact, treatment of HeLa cells with proteasome inhibitors causes the disappearance of clastosomes, supporting the view that proteasome activity is required for their formation (Lafarga et al., 2002a).

**Figure 2:**
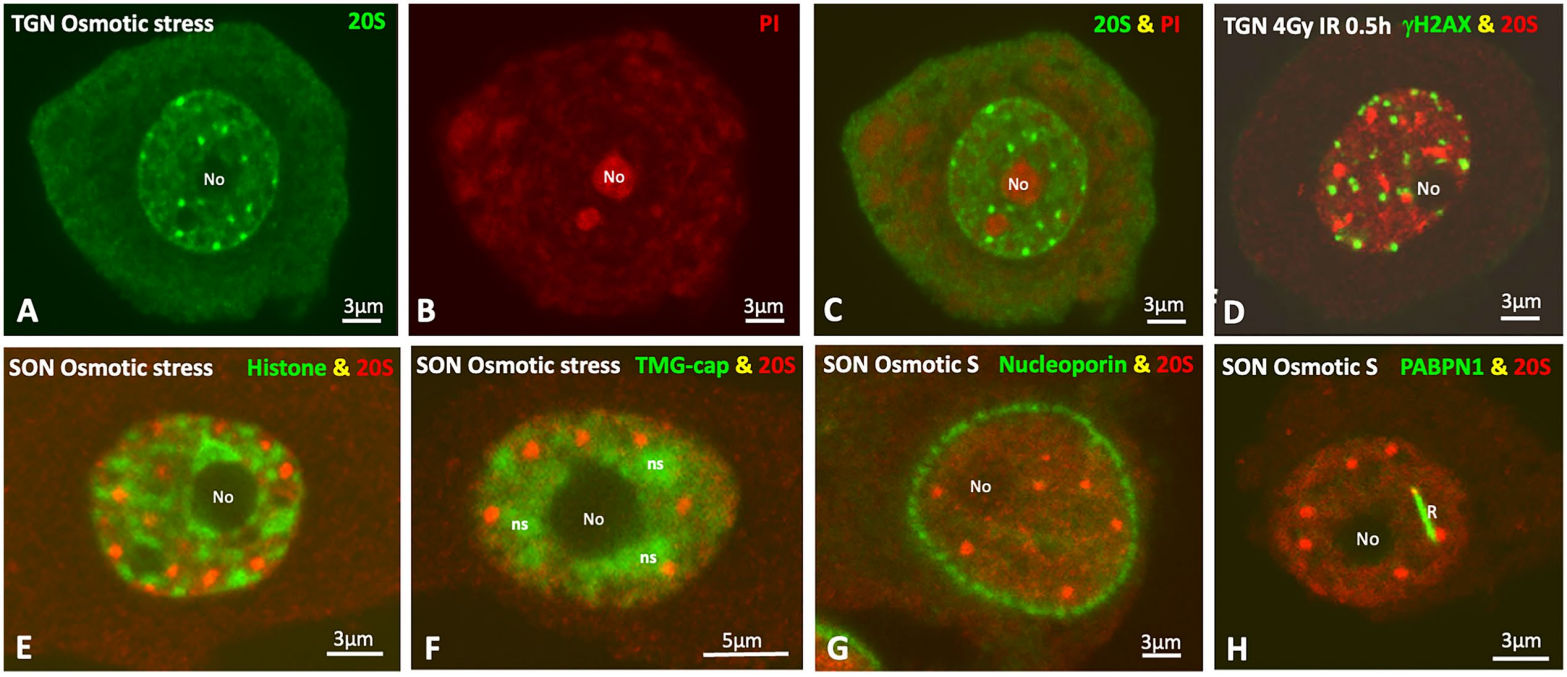
**(A–C)** Osmotic stress-induced formation of clastosomes in rat trigeminal ganglion neurons (TGN). Double fluorescent staining for the 20S proteasome and propidium iodide (PI), a cytochemical staining for rRNA-rich structures. Note the abundance of sharply defined clastosome foci distributed throughout the nucleus and their conspicuous absence from nucleoli and cytoplasm, 3 h after the intraperitoneal injection of a hypertonic NaCl solution. **(D)** DNA-damage-induced formation of clastosomes (red) that do not colocalize with γH2AX-positive DNA-damage foci (green) in a rat TGN (0.5 h after exposure of the rat to 4 Gy X-rays of ionizing radiation) (Figure 2D, from Casafont et al., 2016, Figure 5f, with permission from Springer-Nature). **(E–H)** Osmotic stress-induced formation of clastosomes in rat supraoptic neurons (SON). Double immunofluorescence for the 20S proteasome in combination with pan-histone **(E)**, TMG-cap **(F)**, a marker of nuclear speckles (NS), nucleoporin **(G)**, and PABPN1 **(H)** illustrates the localization of clastosomes in interchromatin regions and their absence from nuclear speckles, the nuclear envelope, and the nucleolus (No). Note in panel H the spatial association of clastosomes with a PABPN1-positive nuclear rodlet (R). Samples were analyzed 3 h after intraperitoneal injection of a hypertonic NaCl solution (Figure 2G, from Carmo-Fonseca et al., 2010, Figure 2C, with permission from Cold Spring Harbor Laboratory Press; Figure 2H, from Villagra et al., 2008, Figure 2I, with permission from Elsevier).

**Figure 3:**
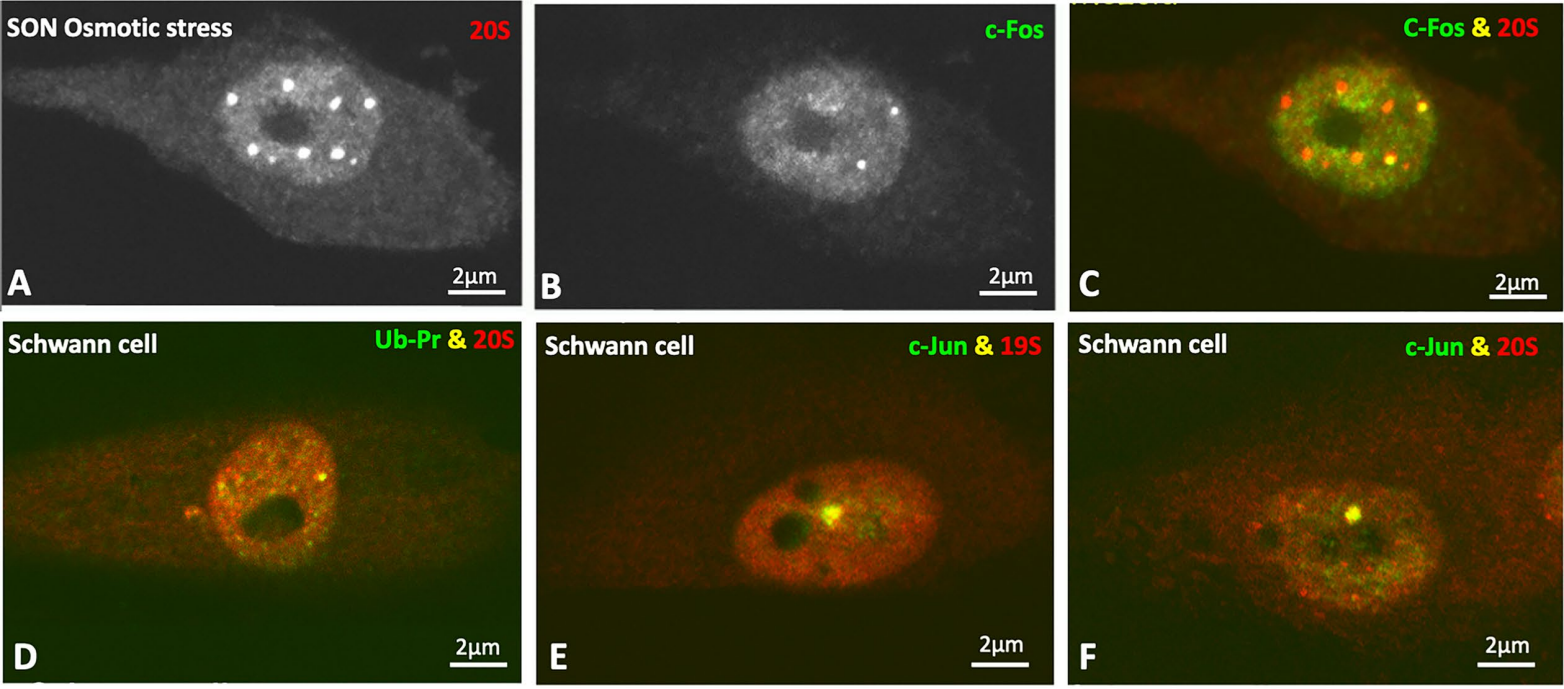
**(A–C)** Double immunostaining for the 20S proteasome and c-Fos in osmotically stressed rat supraoptic neurons (SON), illustrating the formation of multiple prominent clastosomes and nuclear expression of the transcription factor c-Fos. Proteasome 20S and c-Fos colocalize in only two clastosomes, demonstrating their molecular heterogeneity (3 h after the intraperitoneal injection of a hypertonic NaCl solution). **(D–F)** Colocalization in clastosomes of ubiquitin-protein conjugates and c-Jun with 19S and 20S proteasomes in primary cultures of human Schwann cells.

Under basal conditions, clastosomes are relatively sparse. However, their biogenesis is robustly and reversibly induced by osmotic stress in mammalian neurons *in vivo* (Figures 2, 3A–C), highlighting their dynamic nature. Immunofluorescence analysis for detecting molecular markers of nuclear compartments, in conjunction with immunolabeling for 19S or 20S proteasomes, has delineated clastosomes as distinct, generally spherical, nuclear bodies (0.2–1.2 μm in diameter). They reside within the interchromatin space, and are clearly distinguishable from nuclear speckles, Cajal bodies, nucleoli, and nuclear rodlets (Figures 2E–H) (Lafarga et al., 2002a; Lamond and Sleeman, 2003; Spector, 2016; Villagra et al., 2008; Carmo-Fonseca et al., 2010). Furthermore, clastosome biogenesis is transiently observed during the early stages of the DNA damage response in rat sensory ganglion neurons following X-ray irradiation. In this context, they exhibit spatial juxtaposition, though not colocalization, with γH2AX-positive DNA damage foci (Figure 2D) (Casafont et al., 2016).

**Figure 4:**
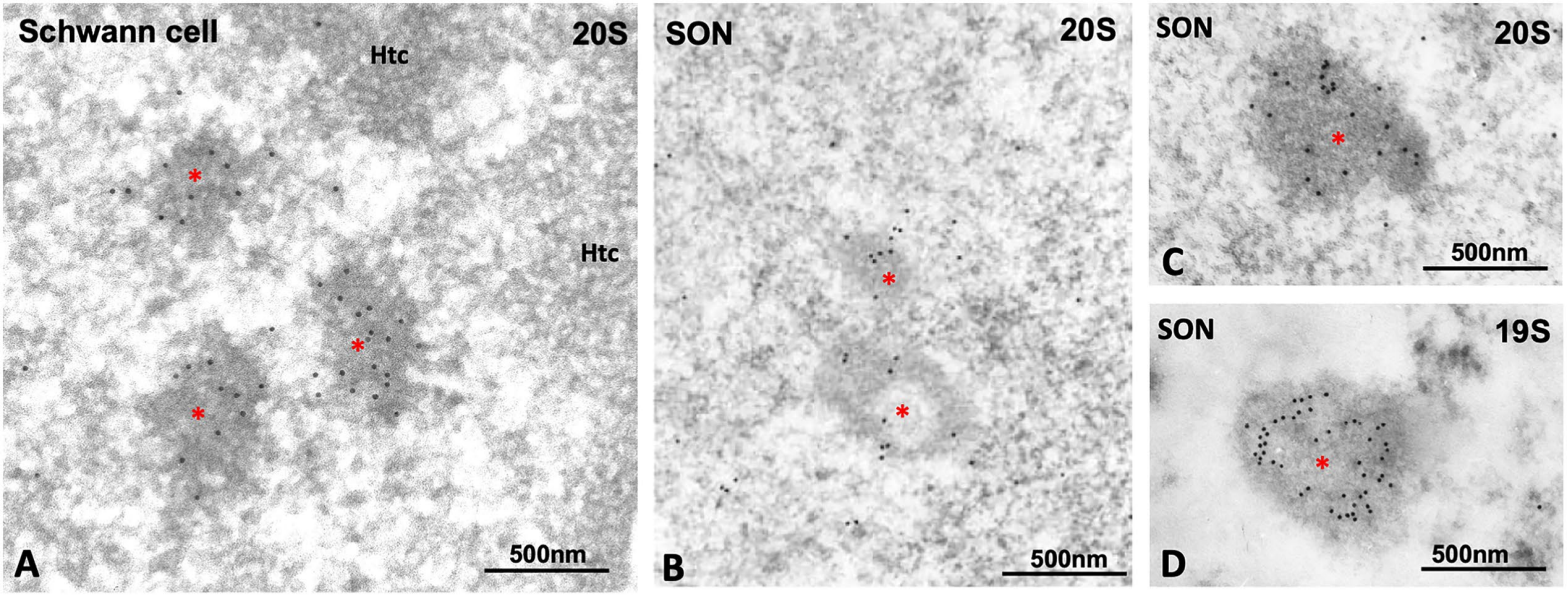
**(A–D)** Immunogold electron microscopy detecting the 20S proteasome in clastosomes from cultured Schwann cells **(A)** and supraoptic neurons (SON) **(B–D)**. Clastosomes appear as membraneless electron-dense nuclear aggregates of rounded or irregular shape decorated with gold particles indicating 20S or 19S immunoreactivity (red asterisks). Note in panel **(B)** a clastosome with a doughnut-shaped profile. No, nucleolus; Htc, heterochromatin masses (Figure 3B, from Carmo-Fonseca et al., 2010, Figure 2E, with permission from Cold Spring Harbor Laboratory Press).

Immunogold electron microscopy has characterized clastosomes as membraneless, electron-dense nuclear bodies (0.2–0.9 μm). They typically appear as partially rounded aggregates (Figures 4A,C,D), although ring-like or doughnut-shaped profiles are also observed (Figure 4B). Clastosomes are stochastically distributed within the interchromatin space and do not maintain stable associations with heterochromatin, nucleoli, or the nuclear envelope (Lafarga et al., 2002a; Carmo-Fonseca et al., 2010).

**Figure 5:**
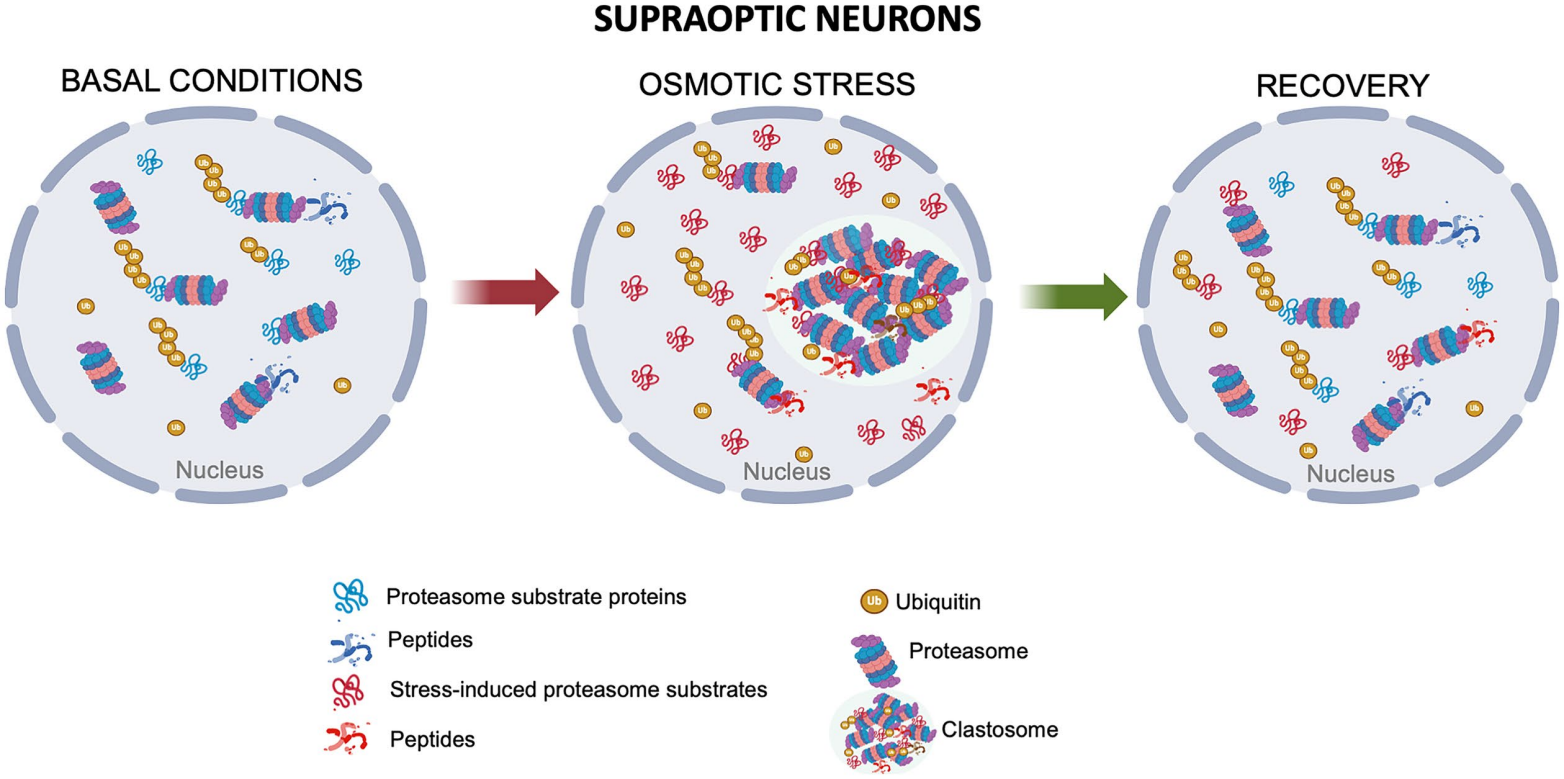
Schematic model illustrating the formation of clastosomes in osmotically-stressed supraoptic neurons *in vivo*. The rapid increase in the nuclear concentration of stress-induced proteasome substrates promotes clastosome assembly. Proteolysis of these proteins can be accelerated when the components of the UPS are concentrated within clastosomes. The proteolytic reduction in the nuclear levels of stress-induced proteins associates with clastosome disassembly and neuronal recovery from stress. Created with BioRender.com.

The functional assembly dynamics of clastosomes was substantiated in mammalian neurons *in vivo* using a hyperosmotic stress model induced by intraperitoneal injection of hypertonic NaCl (Figures 2A–C,E, 3A–C). In hypothalamic supraoptic neurons—osmoregulatory cells responsible for the synthesis of antidiuretic hormone (ADH/vasopressin) (Mecawi et al., 2022)—osmotic stress induces a strong, rapid and transient expression of the transcription factors c-Fos and c-Jun (Lafarga et al., 1992, 1998; Wang et al., 1997). Clastosome frequency peaks approximately 3 h post-stimulation (Figures 2E–H, 3A–C) (Lafarga et al., 2002a). This peak coincides with the accelerated degradation of stress-induced proteasome substrates including c-Fos and c-Jun. Upon cessation of osmotic stress, clastosomes disassemble in parallel with the metabolic clearance of these substrates (Figure 5). Collectively, these observations support the concept that clastosomes form dynamically in response to increased proteolytic demand (Lafarga et al., 2002a; Carmo-Fonseca et al., 2010).

Interestingly, immunolabeling colocalization studies revealed molecular heterogeneity among clastosomes. While some clastosomes within a single neuron sequester c-Fos or c-Jun, others do not, suggesting that distinct clastosome subtypes may exhibit substrate specificity (Figures 3A–C). c-Fos and c-Jun also colocalize with clastosomes in cultured Schwann cells (Figures 3E,F). Additional components reported to localize to clastosomes include ubiquitin conjugates (Figure 3D), the PML protein (Figure 5), adenoviral E1A oncoproteins, and the NO-sensitive guanylyl cyclase β1 subunit (Turnell et al., 2000; Lafarga et al., 2002a; Baltrons et al., 2008; Carmo-Fonseca et al., 2010).

Subsequent studies have identified related nuclear proteasome assemblies in cultured cells under diverse conditions (reviewed in Rockel et al., 2005; Enenkel and Ernst, 2025). These structures have been given multiple names, including FBXO25-associated nuclear domain (FAND) (Manfiolli et al., 2008), proteasome-containing nuclear foci (Yasuda et al., 2020), senescence-associated nuclear proteasome foci (SANPs) (Iriki et al., 2023), p62-containing nuclear condensates (Fu et al., 2021, 2024), and starvation-induced proteasome assemblies in the nucleus (SIPAN) (Uriarte et al., 2021). Advanced methodological approaches have demonstrated that nuclear proteasome foci are biomolecular condensates formed through LLPS (Yasuda et al., 2020; Uriarte et al., 2021; Fu et al., 2021; Iriki et al., 2023). These hubs significantly accelerate substrate degradation by concentrating all necessary UPS components within a single phase (reviewed in Enenkel and Ernst, 2025; Lulu-Shimron et al., 2025).

The seminal work by Yasuda et al. (2020) elucidated the mechanisms of proteasome condensate formation during hyperosmotic stress in HCT116 cells expressing the proteasome subunit PSMB2. The authors provided the first demonstration that the stress-induced proteasome foci exhibit properties of liquid droplets formed by LLPS mechanisms. Using a mass-spectrometry-based proteomic screen of proteasome-associated factors, the authors demonstrated that proteasome foci are enriched in the chaperone p97/VCP, the ubiquitin ligase UBE3A, and the substrate-shuttling factor RAD23B. This factor is a ubiquitin receptor of the UPS that interacts with ubiquitin chains and proteasomes (Walters et al., 2003). Multivalent interactions between K48-linked polyubiquitin chains and ubiquitin receptors such as RAD23B drive condensate formation and proteasome recruitment. Moreover, Yasuda et al. (2020) identified ribosomal proteins as major substrates of proteasome foci. Interestingly, hyperosmotic stress also caused nucleolar stress, a cellular response associated with inhibition of RNA polymerase I-dependent transcription (Boulon et al., 2010; Corman et al., 2023). This nucleolar dysfunction usually leads to the accumulation of ribosome-free ribosomal proteins that are not assembled in nucleolar preribosomal particles (Sirozh et al., 2024), which could then be degraded by nuclear proteasomes. In this context, Lam and colleagues, combining proteomic and imaging analyses, showed that only a fraction of synthesized ribosomal proteins is assembled in preribosomal particles. This finding provided a powerful demonstration of continual proteasomal degradation of unassembled ribosomal proteins in the nucleoplasm (Lam et al., 2007).

In addition to proteasome-containing nuclear foci reported by Yasuda et al. (2020), RAD23B-dependent LLPS facilitates proteasome condensate formation in senescent and starved cells (Iriki et al., 2023; Uriarte et al., 2021). It is also noteworthy that proteolytically active p62-containing nuclear condensates formed by LLPS mechanisms have been identified (Fu et al., 2021, 2024). These condensates have been characterized as proteolytically active sites that increase UPS efficiency, facilitating the degradation of the oncogenic c-Myc and preventing its aberrant nuclear accumulation (Fu et al., 2021; Lulu-Shimron et al., 2025). Interestingly, a recent study reported cytoplasmic p62 condensates that exhibit distinct proteasome substrate preferences compared with nuclear condensates (Lulu-Shimron et al., 2026). While cytoplasmic p62 condensates accelerate degradation of the tumor suppressor p53 and promote tumorigenesis, nuclear condensates stabilize p53 and also promote the degradation of oncogenic c-Myc. Therefore, the subcellular localization of proteolytic p62 condensates directs tumor suppression or growth (Lulu-Shimron et al., 2025).

Taken together, these findings validate and mechanistically extend the concept of the clastosome as a dynamic, membraneless, stress-responsive nuclear proteolytic center, originally proposed by Lafarga et al. (2002a). The molecular composition and spherical shape of clastosomes suggest that they share the basic LLPS mechanisms that drive the formation of the recently characterized nuclear proteolytic centers (Yasuda et al., 2020; Fu et al., 2021, 2024; Uriarte et al., 2021; Iriki et al., 2023). In osmotically stimulated neurons, clastosome formation might be facilitated by the rapid increase in the concentration of stress-induced proteasome substrates, which can exceed a critical threshold leading to phase separation. Moreover, the accumulation of ubiquitin-protein conjugates and substrate proteins with IDRs, such as c-Fos or c-Jun, within clastosomes may promote LLPS. This process is schematically illustrated in Figure 5.

While the clastosome is one specific category of proteasome-enriched nuclear body, there is no conclusive evidence to support the view that recently described nuclear proteasome condensates are the same as clastosomes. In fact, clastosomes become more abundant when proteasomal activity is stimulated and disappear upon proteasome inhibition (Lafarga et al., 2002a). In contrast, the majority of currently known nuclear proteasome condensates increase in number when proteasome activity is inhibited (Yasuda et al., 2020; Fu et al., 2021). Moreover, while clastosomes are formed in mammalian nervous tissue, it remains unclear to what extent the more recently reported nuclear proteasome condensates assemble *in vivo.*

## 5 Association between PML-NBs and clastosomes: the hybrid bodies

PML-NBs are highly dynamic, membraneless nuclear condensates involved in a broad spectrum of nuclear functions, including cellular stress response, genome maintenance, regulation of transcription, DNA damage, antiviral defense, tumor suppression, cell senescence, and apoptosis (reviewed in Zhong et al., 2000; Kumar et al., 2007; Lallemand-Breitenbach and de Thé, 2018). The PML protein—a molecular marker of PML-NBs—serves as the scaffold for PML body assembly into nuclear condensates via LLPS mechanisms driven by multivalent interactions between SUMOylated PML and SUMO-interacting motifs (Shen et al., 2006; Lallemand-Breitenbach and de Thé, 2018; Wu et al., 2023; Fu et al., 2024). These condensates contain PML-body interacting proteins, including SP100, DAXX, p53, SUMO 1/2/3 and the E3 ubiquitin-protein ligase RNF4, and do not accumulate ribonucleoproteins (reviewed in Lallemand-Breitenbach and de Thé, 2018).

Structurally, PML-NBs generally consist of a peripheral shell of PML protein that encapsulates an inner core enriched in SUMOylated client proteins. There is significant morphological and molecular heterogeneity among PML-NBs, given rise to various PML-NB subtypes. Moreover, a fraction of the PML protein remains diffusely distributed throughout the nucleoplasm (reviewed in Lallemand-Breitenbach and de Thé, 2018; Lang et al., 2010).

Importantly, in certain mature stages of PML-NBs, and especially upon treatment with arsenic trioxide, the PML protein itself can be targeted for proteasomal degradation through SUMO-targeted ubiquitylation mediated by RNF4 (Lallemand-Breitenbach et al., 2001, 2008). This E3 ubiquitin-protein ligase induces the formation of ubiquitin chains on polySUMOylated PML protein, thereby targeting it for proteolytic clearance by the proteasome (reviewed in Lallemand-Breitenbach et al., 2008; Tatham et al., 2008; Fu et al., 2024). Supporting the role of these bodies as sites of intranuclear proteolysis, both the 11S regulatory subunit and the 20S catalytic core have been localized within these structures (Lallemand-Breitenbach et al., 2001).

**Figure 6:**
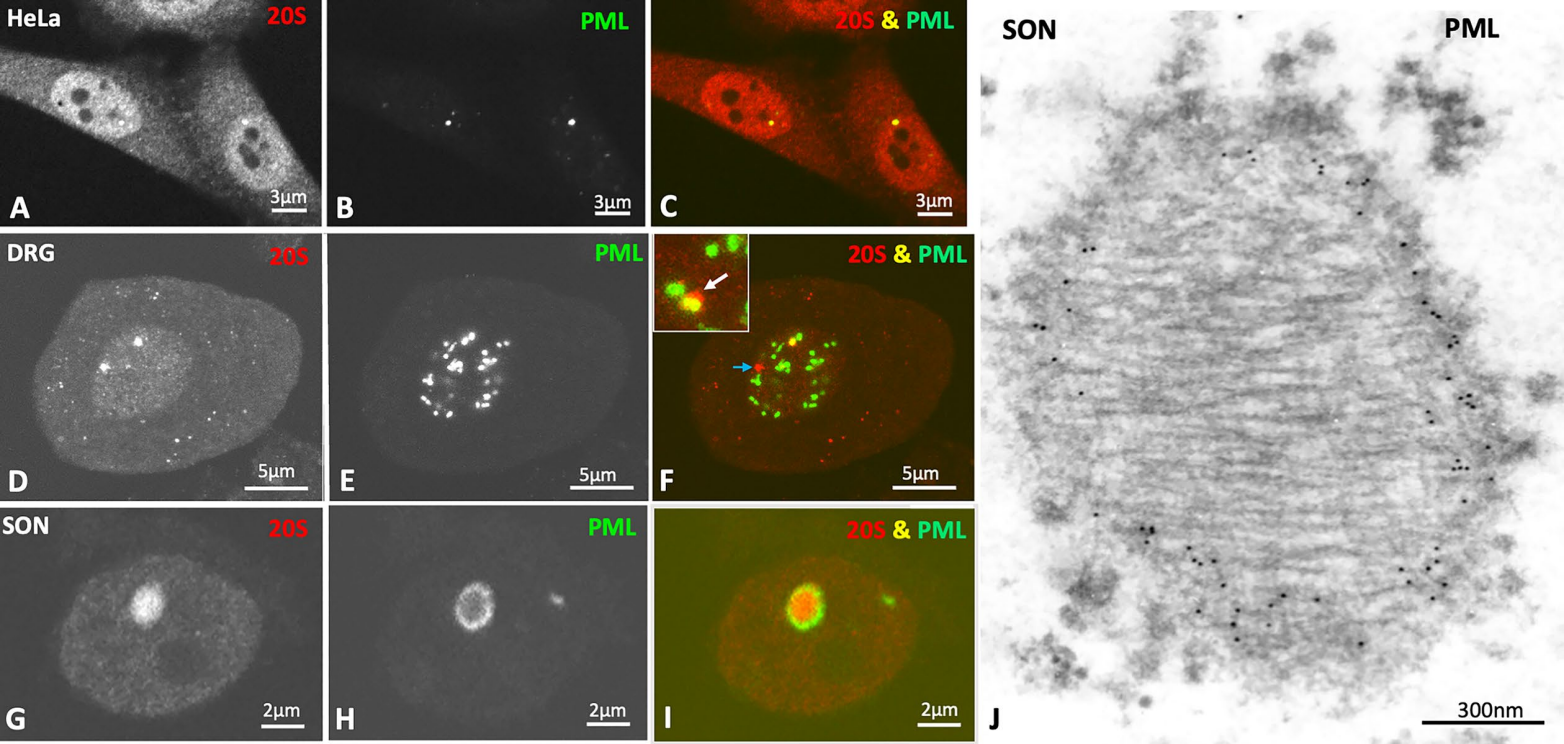
**(A–J)** Double immunolabeling for PML protein and the 20S proteasome in cultured HeLa cells **(A–C)** and human dorsal root ganglion (DRG) **(D–F)** and supraoptic (SON) **(G–J)** neurons. **(A–C)** Note the complete colocalization of PML protein and the 20S proteasome in clastosomes from HeLa cells. **(D–F)** This DRG neuron contains an abundance of PML bodies free of proteasomes, a 20S-positive clastosome (blue arrow), and another clastosome showing partial colocalization of PML protein (Inset, white arrow) (Figures 5D,F from Carmo-Fonseca et al., 2010, Figures 3A,B, with permission from Cold Spring Harbor Laboratory Press). **(G–I)** A PML body in a SON neuron with a typical peripheral shell enriched in PML-positive protein and an inner core intensely immunolabeled for the 20S proteasome and poor in PML protein. **(J)** Immunogold electron microscopy detecting PML protein in the peripheral layer of a PML body from a SON. Note the absence of gold particles in the inner core (Figures 5G–J, from Villagra et al., 2006, Figures 4M–O and 5B, with permission from Elsevier).

While PML-NBs and clastosomes are clearly distinct nuclear structures, different forms of interactions between clastosomes and PML-NBs have been reported (Lafarga et al., 2002a; Janer et al., 2006; Carmo-Fonseca et al., 2010; Villagra et al., 2006). Indeed, the colocalization of PML protein and proteasomes has been observed in specific nuclear bodies in HeLa cells (Figures 6A–C) (Lafarga et al., 2002a; Carmo-Fonseca et al., 2010). In human dorsal root ganglion neurons from patients without neurological pathology, the majority of PML-NBs—immunoreactive for the PML protein—lack proteasomes, while some proteasome-rich clastosomes assemble independently of PML-NBs (Figures 6D–F). Importantly, in this neuronal population, we have found the spatial association, resembling docking, between PML-NBs and clastosomes forming hybrid bodies (Figure 6F) (Carmo-Fonseca et al., 2010). A different form of attachment of the hybrid body has been found in human supraoptic neurons without neurological involvement, in which a proteasome-rich inner core appears completely encapsulated by a shell enriched in PML protein (Figures 6G–J) (Villagra et al., 2006). Collectively, these findings support a dynamic interplay between clastosomes and PML-NB condensates. Consistent with this view, recent studies in cultured cells have demonstrated the formation of hybrid bodies between stress-induced p62 nuclear bodies and PML-NBs (Souquere et al., 2015; Fu et al., 2024). Particularly, Fu et al. (2024) have reported that the interactions between PML-NBs and p62 nuclear condensates do not lead to fusion or mixing of their contents, but rather result in the translocation of RNF4 from PML-NBs to the p62 condensate. This mechanism stabilizes PML-NBs and potentially may enhance their multiple functions for maintaining cellular health (Fu et al., 2024).

The interaction between PML-NBs and clastosomes in neurodegenerative disorders will be discussed in the following section.

## 6 PML NBs-clastosomes interplay in neurodegeneration

The nuclear accumulation and subsequent aggregation of misfolded or mutant proteins represent a hallmark pathogenic mechanism in numerous neurodegenerative disorders (reviewed in Ciechanover and Brundin, 2003; VerPlank et al., 2018; Guo et al., 2014; Zbinden et al., 2020). Postmitotic neurons are particularly susceptible to these proteinopathies, as they lack the capacity for mitotic dilution—the process by which dividing cells reduce the burden of damaged proteins through the breakdown and reformation of the nuclear envelope. To counteract the accumulation of aberrant proteins, neurons activate two primary cytoprotective mechanisms: the refolding of polypeptides into their native conformations by molecular chaperones and co-chaperones, and their targeted elimination via degradative pathways, most notably the UPS (Goldberg, 2003; Guo et al., 2014).

In neurodegenerative proteinopathies, UPS dysfunction—caused by disease protein overload or inhibition by protein aggregates—is considered a key driver of nuclear aggregates containing misfolded or mutant proteins (Ciechanover and Brundin, 2003; Janer et al., 2006; Bennett et al., 2007; Chort et al., 2013; Dantuma and Bott, 2014; Guo et al., 2014). This suggests that failure of clastosome-mediated nuclear PQC may represent a convergent pathogenic mechanism. However, whether toxic protein aggregation is protective—by promoting UPS-dependent degradation within discrete nuclear domains—or instead contributes to neuronal dysfunction remains unclear. In the latter case, toxic proteins may sequester within aggregates essential factors for nuclear functions, resulting in widespread transcriptional dysregulation and dysfunction of mRNA processing (Woulfe, 2008; von Mikecz, 2014; Pinho et al., 2019).

An important question in neurodegenerative proteinopathies is the relationship between PML-dependent SUMOylation and UPS-based protein degradation pathways. While the PML-NB was especially studied in cancer cells, acting as a tumor suppressor, it has important functions in other cell types such as neurons. Thus, neuronal PML-NBs appear to have multiple functions, including neuronal differentiation, transcriptional regulation of genes involved in synaptic plasticity, circadian rhythms, and the response to toxic proteins that cause neurodegeneration (reviewed in Korb and Finkbeiner, 2013; Wagner et al., 2025; Wang et al., 2026).

Polyglutamine (polyQ) diseases—including Huntington’s disease, spinocerebellar ataxias (SCAs), spinobulbar muscular atrophy, and dentatorubral-pallidoluysian atrophy—provide a suitable system for studying the involvement of PML-NBs and clastosomes in the clearance of toxic protein aggregates. These proteinopathies are characterized by pathological CAG repeat expansions that drive nuclear aggregation of mutant proteins (Takahashi et al., 2002, 2003; Shao and Diamond, 2007; Nguyen et al., 2025; Villavicencio-Gonzalez and Zoghbi, 2026).

In particular, spinocerebellar ataxia type 7 (SCA7), caused by CAG expansion in the *ATXN7* gene, serves as a prototypical model for these processes. The encoded protein, ataxin 7, is a transcriptional regulator involved in histone H2B ubiquitylation and H3K9 acetylation (Mohan et al., 2014). Mutant ataxin 7 accumulates in neuronal nuclei, forming inclusions that contain chaperones, proteasome subunits, PML protein, and SUMOylated proteins (Takahashi et al., 2003; Janer et al., 2006, 2010). These findings support a functional link between PML-NBs and clastosome-related proteolytic mechanisms mediated by the PML-dependent SUMOylation of mutant ataxin-7 (Janer et al., 2006; Chort et al., 2013; Marinello et al., 2019). These studies by Annie Sittler’s team demonstrated in cell lines and primary cultures that overexpression of PML isoform IV results in the assembly of nuclear bodies that the authors refer to PML clastosomes (Janer et al., 2006; Chort et al., 2013; Marinello et al., 2019). Moreover, these PML clastosomes recruit soluble mutant ataxin 7 for degradation through SUMO-targeted ubiquitylation mediated by the E3 ubiquitin ligase RNF4 (Janer et al., 2006; Marinello et al., 2019). In SCA7 knock-in mice, interferon beta upregulates PML protein expression and increases the number of PML clastosomes, resulting in enhanced degradation of mutant ataxin 7 (Chort et al., 2013). These effects mitigate neurodegeneration by improving animal performance on behavioral tests (Chort et al., 2013). In addition to mutant ataxin 7, these authors demonstrated the degradation within PML clastosomes of aggregation-prone huntingtin exon 1 carrying 125 polyglutamine repeats, suggesting that interferon-beta-induced degradation of mutant ataxin 7 could also be effective in other polyglutamine disorders (Janer et al., 2006; Chort et al., 2013). Similarly, the involvement of SUMOylation-ubiquitylation networks in the disaggregation of pathogenic nuclear aggregates has been analyzed in other neurodegenerative disorders, including polyG, polyGA and TDP-43 proteinopathies (Wagner et al., 2025; Wang et al., 2026).

In neuronal populations that constitutively contain clastosomes, a reduction in clastosome frequency may signify a failure in nuclear PQC, potentially precipitating neurodegeneration. For instance, a murine model of Down syndrome (DS) exhibited a significant reduction in clastosome number per nucleus in hippocampal granule cells (GCs), accompanied by a 35% decline in hippocampal proteasomal activity (Puente-Bedia et al., 2022). This suggests that these neurons possess an insufficient degradative capacity to maintain nuclear proteostasis. Given the gene dosage imbalance in DS, these GCs also display epigenetic dysregulation and a diminished global transcriptional rate, as evidenced by *in situ* 5′-fluorouridine assays (Puente-Bedia et al., 2021). In DS, while it is evident that gene dosage effects contribute to UPS disruption (reviewed in Tramutola et al., 2026), it remains to be determined whether this transcriptional depression directly impacts the expression of proteasome-related genes, thereby hindering clastosome assembly. Notably, recent studies in Alzheimer’s disease have identified intrinsic alterations in proteasome complexes linked to the downregulation of essential transcriptional regulators (Jiang et al., 2025).

When proteasomal activity is impaired, a compensatory mechanism may involve the CRM1-mediated nuclear export of ubiquitinated proteins to the cytosol to maintain nuclear proteostasis (Hirayama et al., 2018). Once in the cytoplasm, these proteins tend to form insoluble aggregates (Hirayama et al., 2018; Xu et al., 2023; Moors et al., 2024). This shift in nucleocytoplasmic partitioning of ubiquitin-protein aggregates—from nuclear clastosome-associated foci to cytoplasmic inclusions—has been observed in the GCs of this model of DS (Puente-Bedia et al., 2022). Moreover, the accumulation of APP, Aβ, hyperphosphorylated Tau, and oxidized proteins in the DS hippocampus (García-Cerro et al., 2017; Parisotto et al., 2016) may sequester available proteasomes, further limiting their nuclear import and subsequent clastosome biogenesis.

## 7 Concluding remarks and future perspectives

In 1910, the seminal investigations of Cajal established the structural foundations of nuclear organization in mammalian neurons (reviewed in Lafarga et al., 2025). More than a century later, the advent of advanced imaging and multi-omics technologies has profoundly expanded our understanding of the nuclear landscape, facilitating the characterization of novel nuclear bodies now recognized as nuclear condensates.

Within the broader framework of the nuclear UPS, the clastosome emerges as a clearly distinct nuclear body. The term clastosome provides a precise, historically grounded, and functionally descriptive nomenclature for a specialized, nuclear-specific proteolytic compartment. In fact, clastosomes promote nuclear proteolysis by concentrating UPS components, proteasome substrates and chaperones in a discrete nuclear domain (Figure 5). However, one major challenge is the precise molecular definition of clastosomes and their distinction from other recently described nuclear proteasome condensates.

The dynamic nature of the assembly/disassembly cycle of clastosomes in response to cellular stress supports their important cellular function by enhancing nuclear protein turnover and PQC. This role is particularly relevant in neurons, which must maintain proteome integrity throughout their lifetime. In general, it appears that activation of clastosome assembly may have a beneficial effect in the recovery from cellular stress and presumably in other proteostasis disturbances. Several key questions remain open, including the molecular mechanisms, biophysical principles and specific signals underlying clastosome biogenesis and dynamics. Further investigation is also required to elucidate whether clastosome formation, turnover, and functions may differ across cell types in living tissues under physiological, or under different experimental and pathological conditions. Of particular relevance is the relationship between PML-NBs and clastosomes, which appears to be mediated by the intersection of the SUMOylation and ubiquitin-proteasome degradation pathways. This functional interaction appears to be involved in the clearance of nuclear aggregates in certain neurodegenerative proteinopathies. However, the precise molecular mechanisms underlying these beneficial effects remain to be fully defined.

From a translational perspective, understanding the mechanisms regulating clastosome formation and activity may open new therapeutic avenues for neurodegenerative diseases and other disorders associated with impaired proteostasis. Enhancing nuclear proteolytic capacity, promoting protective proteasome-condensate assembly, or modulating SUMO–ubiquitin signaling pathways could facilitate the clearance of toxic nuclear proteins. It remains to be determined whether clastosome dysfunction is not only a consequence but also a driver of neurodegenerative disorders.

By revealing the stepwise clearance of pathogenic nuclear aggregates, recent studies suggest the functional link between SUMOylation and UPS-mediated degradation as an important regulator of nuclear proteostasis. Although the therapeutic relevance of these findings remains to be determined, they provide proof of principle that targeting PML-NB-clastosome interplay may offer a viable therapeutic strategy in neurodegenerative proteinopathies.

Current studies on the function of a specific nuclear proteolytic center, the p62 nuclear body, in cancer cell lines support the view that this nuclear condensate may act as a tumor suppressor by promoting the degradation of the oncogenic c-Myc (Fu et al., 2024). These findings open a new horizon for investigating the potential role of clastosome activation in restoring proteostasis in tumor cells in animal models of carcinogenesis.
